# A Review and Analysis of the National Dog Population Management Program in Chile

**DOI:** 10.3390/ani12030228

**Published:** 2022-01-19

**Authors:** Elena Garde, Paula Marín-Vial, Guillermo E. Pérez, Erik M. Sandvig

**Affiliations:** 1 Global Alliance for Animals and People, Pasaje Los Arrayanes 333, Valdivia 5110624, Chile; paula.marin@thegaap.org (P.M.-V.); guillermo.perez@thegaap.org (G.E.P.); 2 Instituto de Ecología y Biodiversidad, Departamento de Ciencias Ecológicas, Facultad de Ciencias, Universidad de Chile, Las Palmeras 3425, Ñuñoa, Santiago 7800003, Chile; erik.sandvig.c@gmail.com

**Keywords:** free-roaming dogs, dog population management, Chile, responsible dog ownership, sterilization, humane education, dog census, human-to-dog estimates, identification and registration

## Abstract

**Simple Summary:**

Free-roaming dogs are a worldwide problem that cause a wide array of issues in society. In Chile, a law protecting companion animals, and describing the role of responsible owners, was promulgated in 2017, followed by a national program aimed at mitigating dog-related problems throughout the country. The aim of this review is to explain and discuss certain components of the program and to provide suggestions for future changes. A simple model was created to assist managers in Chilean municipalities to more closely estimate the local dog population. The recommendations include the collection of baseline data and a dog population size estimate prior to implementing interventions, as well as the development of clear objectives, achievable goals and indicators, and the investment of public funds in long-term sustainable strategies such as education and enforcement rather than offering free veterinary services.

**Abstract:**

Free-roaming dogs are a worldwide problem, with Chile having some of the highest human-to-dog ratios in the world. In 2017, Law 21.020 was promulgated and the federal government developed a national responsible pet ownership program. The objectives of this article are to describe and discuss the dog-related components of the program, to design a tool for determining human-to-dog ratios in Chile, and to make recommendations to managers to improve the program outcomes. The overarching goal of the program was to mitigate the conflict between humans and dogs, but many of the interventions were animal-focused and the indicators did not consider the perception of the Chilean public. Using human density data and known dog populations, we found that as the human density increased, there were fewer dogs per person. Veterinary services and sterilizations were the mainstay of the program and were offered for free to citizens. Education was offered to all ages through public events, as well as municipality and organization activities. The identification of dogs was obligatory for dog owners. Enforcement was not included in the program. The recommendations are to conduct preintervention baseline data collections and to tailor interventions and indicators appropriately; to use dog population size estimates determined at the local level rather than a country-wide estimate; to replace free veterinary services with low-cost sterilization campaigns; to create sustainable plans for education; and to create enforcement teams in communities.

## 1. Introduction

Free-roaming dogs (FRDs) are a worldwide problem, particularly in developing countries [1,2,3,4]. Dogs that are allowed to roam unsupervised cause an array of problems such as vehicle accidents, dog fights, disease transmission, attacks on wildlife, attacks on other domestic animals and humans, uncontrolled reproduction, and the contamination of public areas with fecal matter and garbage [1,2,3,5,6,7].

There are a number of international guidelines from organizations such as the World Organization for Animal Health (OIE, Paris, France) [8] and the International Coalition for Animal Management (ICAM, Cambridge, UK) [9], as well as a number of published articles that offer detailed recommendations for dog population management (DPM) in countries facing the socioeconomic, environmental, political, religious, and human and animal health impacts of FRDs [1,2,3]. The responsibilities of government and non-government organizations (NGOs) are well-delineated in these recommendations and the guidelines provide clear steps towards the formation of advisory groups, pre- and post-data collection, a regulatory framework, and recommendations for a number of program elements, including baseline data collection, to gain a clear appreciation of the local need. This includes a dog population estimate, as well as education, legislation, reproduction control, identification, and registration. These documents also include a monitoring and evaluation framework, as without it there is no ability to measure performance and accountability.

It is important to note that although these guidelines exist, there is no “one size fits all” formula to follow to solve the FRD issue. Countries and communities are all unique and require a tailored approach depending on the local situation. Sadly, many countries spend years investing in DPM programs, resulting in disappointing results, often because the approach is generic or unrelated to the issues in situ or because the execution is scattered and uncoordinated [6].

While FRDs in Chile have been identified as a national, sociocultural, and animal welfare issue for decades [10,11], the release of the responsible pet ownership law and a complementary national pet/companion animal plan has only occurred within the last five years.

Chile, a country in South America with 17,574,003 inhabitants [12], has gained national and international attention over the last few decades because of the various economic, animal welfare, environmental, and social problems caused by FRDs. This is somewhat scandalous because Chile is considered one of South America’s most prosperous nations, with stable long-term economic growth [13]. It is a country known for its beautiful landscapes that are home to endemic wildlife species [14] and whose survival is threatened by dog attacks and/or diseases transmitted by FRDs [15,16,17]. Chile is also a country dependent upon agriculture, with livestock production being one of its main industries [18]. There are almost four million sheep in the country, with 64% of those originating from large herds in Patagonia, while the remainder are from smaller herds of 50 to 500 from the south-central part of the country [19]. For many of these flocks, dog attacks are the number one threat to production and a problem that has driven many small producers out of the industry [20].

Although Chile does not have canine rabies, a host of canine zoonoses [7,21,22] and dog bites [23] have had significant public health impacts. Dogs have also made their mark in online tourism blogs with a surprising number of articles, comments, and stories written by visitors to the country remarking on the astonishing number of dogs seen wandering every city of the country [24,25,26]. While most tourists seem to find the dogs well-fed and friendly, according to studies most Chileans consider FRDs to be a chronic and growing problem in their neighborhoods and communities [27,28].

In 2006, household surveys were done in Chile to evaluate factors influencing the quality of life of the citizens. They found that 50.4% of citizens said that conflicts associated with the presence of free-roaming dogs and cats were the number one problem that should be prioritized in their communities. In 2015, this number had dropped to 48.2%; however, in that same survey, 63% of respondents stated that FRDs constituted a serious or very serious problem. Then, in 2018, the survey was repeated and the majority of respondents said that FRDs and other animals were the most serious problem in their neighborhoods, surpassing even that of crime [29].

Municipalities are given the lion’s share of the responsibility for managing the country’s FRD problem. However, in many cases there is an insufficient capacity or insufficient staffing to deal with such a complex issue, or there are limited economic resources to allocate to baseline data collection. Therefore, a clear analysis of the local situation is unavailable. Yet, published data suggest that Chile differs markedly between regions in terms of dog ownership practices, such as the numbers of dogs that residents own, emphasizing the importance of understanding the human–canine dynamic before implementing new interventions. A lack of clarity about these dynamics and the ensuing problems on a site-by-site basis can result in programs with unclear objectives, ineffective interventions, or a lack of coordination from municipality to municipality [30,31].

The objectives of this article are: (1) to describe and discuss general components of the national companion animal management plan as it pertains to dogs only, with a particular emphasis on veterinary services, education in responsible pet ownership, and registration and identification; (2) to provide a tool for determining the human-to-dog ratios in Chile for their use in municipalities where a specific dog population estimate is not available; and finally, (3) to make recommendations for some adaptations to the strategies and indicators of the DPM plan that could potentially improve the intervention outcomes and the long-term sustainability of the program.

## 2. The Background of the Responsible Pet Ownership Law

**Figure 1:**
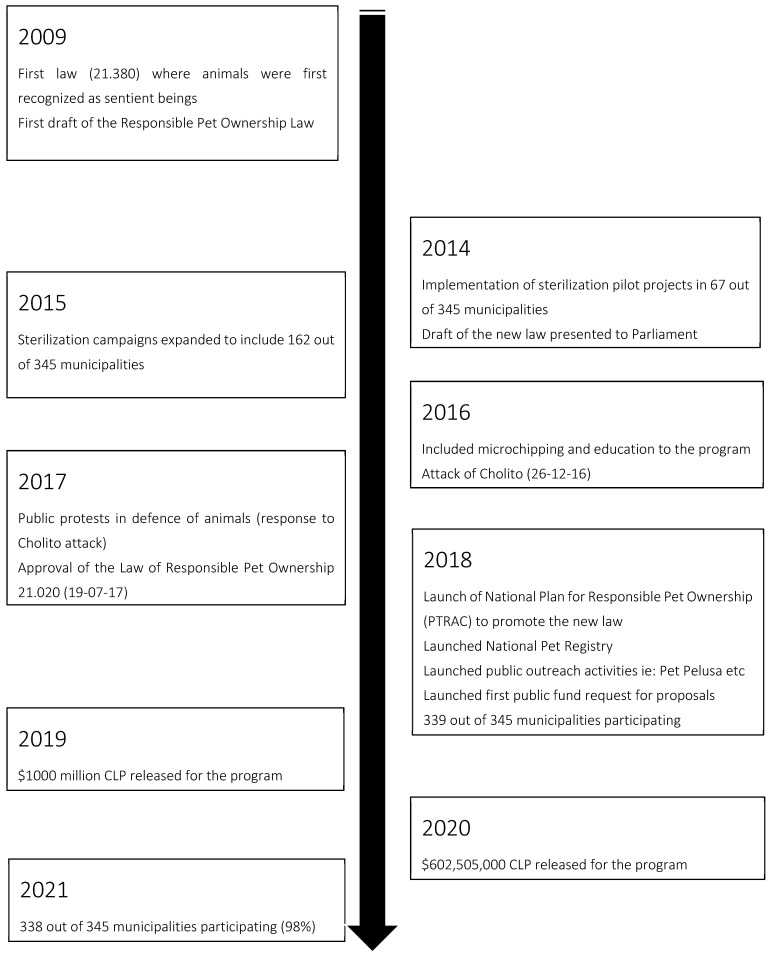
Timeline of important landmark events in the formation and implementation of the National Program for the Responsible Ownership of Companion Animals (PTRAC in Spanish: Programa Tenencia responsible para Animales de Compañía) between 2009 and 2021.

The first draft of the responsible pet ownership bill dates back to 2009 and primarily describes the liability for damages caused by potentially dangerous animals. The bill remained in Congress for years, during which time the scope was amplified to include content related to general pet ownership. In the meantime, in 2014 the government released funds for a pilot sterilization program in 67 (19%) municipalities and then, in 2015, in a further 162 (46.9%) out of a total of 345 municipalities in the country. In 2016, the program expanded to include the microchipping of pets and public outreach about the responsible ownership (RO) of cats and dogs [29]. Then, in December of 2016 while the 2009 bill was still in parliament, the year closed with the brutal attack and presumed death of the now locally famous street dog “Cholito” [32]. The attack occurred in the Patronato neighborhood in the city of Santiago by two individuals with sticks and was filmed by a passerby; the video was subsequently uploaded to the internet, provoking a massive protest by indignant and outraged Chileans demanding that companion animals be offered more protection under the law against abuse, neglect, and mistreatment. The movement termed #justiceforcholito accelerated the promulgation of the Responsible Pet Ownership Law 21.020, also called the “Cholito Law”, which was finally approved in July 2017 (Figure 1).

The Cholito Law, or the Responsible Pet Ownership Law (Law 21.020), complements the 2009 “Law on the Protection”of Animals” (Law 20.380) which, although it originally did state that animals are sentient beings and deserving of protection, was deficient in details about how to comply with the law and also continued to consider animals as personal property. The new law (Law 21.020), aims to generate a social and cultural change in the way that citizens interact with companion animals and describes a set of obligations that a person agrees to when he or she decides to keep a pet; that is, to provide it with food, a home, and to treat the pet in an appropriate manner, as well as providing them with veterinary care and not subjecting them to suffering or abandonment in addition to respecting public health and safety regulations [33]. The new legal framework includes three main pillars of action: appropriate animal health care through free municipal-run sterilization campaigns and an increased access to veterinary services; pet identification and registration; and the education of the public about responsible pet ownership.

In 2018, following the release of Law 21.020, the government launched the National Program for Responsible Ownership of Companion Animals (PTRAC in Spanish: Programa Tenencia Responsable para Animales de Compañía), hereafter referred to as PTRAC. Municipalities and NGOs working in the field of veterinary medicine, welfare, and/or responsible pet ownership, and who registered in a National Registry, were permitted to apply for public funds to implement the PTRAC [29]. From 2014 to 2021, CLP 30,193,511,206 (approximately USD 38 million) was allocated to the municipalities [34] to implement PTRAC and 338 of 345 municipalities participated, corresponding to 98% of the total number of districts in the country. Additionally, from 2018 to 2021 inclusively, NGOs have been awarded CLP 2,155,838,605 (approximately USD 3 million) [34] to assist with the implementation of PTRAC. A total of more than 2300 municipal and NGO projects have been financed nationwide.

## 3. The Overview of the Chilean National Program for the Responsible Ownership of Companion Animals

### 3.1. Objectives and Indicators

The overarching goal of the PTRAC was centered around the conflict between people, companion animals, and animal welfare. The main objective was to contribute to a better quality of life for all the citizens of Chile by promoting the appropriate coexistence between people and animals, as well as having people live responsibly with companion animals [29]. Many of the specific objectives were detailed in the PTRAC, such as the to improvement of animal health and welfare, increasing animal rights, reducing dog-related public health risks, and increasing RO. However, some of the main program activities, such as the sterilization campaigns which were the backbone of the program, did not have clear goals in communities where they were executed, nor did they have justification for the implementation of the campaigns.

In order to measure the progress of the program and evaluate for success, seven indicators were created that were related to program performance:

1. The average age of sterilizations
2. The percentage of the coverage of animals accessing veterinary services through, exclusively, municipal clinics for the first time
3. The percentage of the coverage of municipalities
4. The rate of change of the number of illnesses that animals present
5. The percentage of municipalities that continued the program after national funding with their own resources
6. The rate of change in the number of infractions of the Responsible Ownership Law
7. The rate of change of dogs that are free-roaming

These indicators, however, have no associated timelines to assess temporal progress or clear indications of what the expected outcome was. While the main goal was to improve the quality of life of the Chilean public, there are no human-related indicators to specifically address this goal; rather, the indicators are focused on indices related to veterinary care, coverage, fines, or the number of FRDs, for example [29].

### 3.2. The Dog Population Estimate

Based on the literature, there is a very wide range of reported ratios of humans and dogs in Chile, ranging from 10.7:1 [35] to 1.1:1 [5], clearly indicating that, for management purposes, it is critically important to either perform a local census or have some other means of determining a population estimate that closely represents the local reality. With no site-by-site surveys or censuses done to determine the overall dog population, the ratio and calculated estimate for the whole country was based on the results of household surveys conducted in Santiago, Chile [29,36,37], as well as the 2017 national census, to arrive at a final ratio of humans to owned dogs of 5.1:1, regardless of whether the communities were rural or urban. There is currently no estimate of the total population of dogs, including confined dogs, as well as owned and unowned FRDs. It is unusual for a ratio of the number of humans compared to the number of dogs to be consistent and relevant across an entire country, considering socioeconomic, cultural, and lifestyle variations from cities, to towns, to villages. Indeed, the astonishing variability of reported ratios in the published literature from Chile [5,35,36,38] confirms that a single ratio is not useful for DPM across the whole country.

There have been many articles that describe the different methodologies for calculating a dog population estimate [38,39,40]. This is a vital step in any DPM program, and it is important that the estimates are then used to aid in goal setting for the interventions. For example, calculated dog population estimates can be used to determine how many sterilizations should be done, how many dogs should be registered, and how many dogs should be vaccinated to attain herd immunity against important diseases.

Understandably, every small community may not have the economic means or expertise to conduct a household survey or a canine census; therefore, given the importance of having a dog population estimate prior to the commencement of any interventions, we developed an alternative tool for estimating the ratio based on the local human density.

#### 3.2.1. Statistical Analysis

We fit a linear regression model to the ratio of the number of humans, compared to dogs, as a function of the human density at the municipal level (known as a *comuna* in Chile). A dataset based on previous work by Astorga et al. and other recently published papers [35,36,41,42] was collated and filtered to only include studies conducted from the year 2000 and later. Only those that measured the ratio of humans to dogs at the municipal level were included. When studies reported multiple ratio estimates within a municipality (e.g., separate estimates for urban and rural areas or multiple smaller communities within a municipality), estimates were averaged to relate to the density at the municipality level. Human population numbers and the derived densities were taken from national census data. We used census data that was closest to the year of each study. The human density data was log transformed in the model to conform to assumptions of normality. Analyses were done in R [43] and linear regressions were fit using the package *lmer* [44]. The goodness-of-fit of the model was assessed by calculating the adjusted R^2^ (0.152), as well as by examining the distribution of the residuals, which were found to be randomly distributed. In a post-hoc test comparing the performance between a model using the full dataset and a model excluding a single outlying data point at a very high density, we found a 9% improvement in the accuracy of the prediction. Because the aim of the model was to provide a more accurate means to estimate the human-to-dog ratio, we only reported the results for the model excluding the outlier data point.

#### 3.2.2. Results of Model

**Figure 2:**
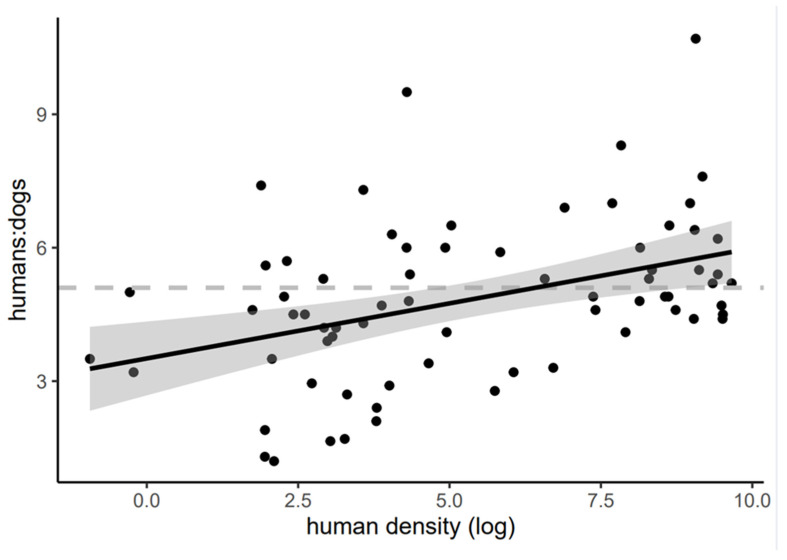
Linear regression (black line) between the ratio of the number of humans to dogs per municipality as a function of human density in that municipality (*n* = 71). Gray area depicts the 95% confidence interval. Grey dashed line refers to the 5.1 human-to-dog ratio commonly used as a baseline in the national program.

We found strong evidence of a negative relationship between human density and the ratio of humans to dogs in Chilean municipalities (slope = 0.248, *p* = 0.0005). As human density increased, for example in urban areas or cities, the number of dogs per human decreased. Conversely, in rural areas with a lower density of people per square kilometer, there were more dogs per human (Figure 2). By dividing the residual standard error of the model (1.663) by the mean of the human-to-dog ratio in our dataset (4.844), we can say that the log of the human density in a municipality accurately predicts the human-to-dog ratio with a 34% error.

The first model described above can be used to calculate a reasonable estimate of the human-to-dog ratio for a municipality in Chile by inserting the human density of the municipality in the following equation. Here, y is the human-to-dog ratio and β is the human density in the municipality.

(1) y=0.248∗log(β)+3.510

### 3.3. PTRAC Strategy 1: Veterinary Services

The veterinary services strategy of the PTRAC is comprised of multiple interventions, including surgical sterilizations, microchipping, preventive medicine such as vaccinations and parasite control, the construction of municipal veterinary clinics, the hiring of additional municipal veterinarians, rescuing and rehoming street dogs, and the care of animals in community shelters.

In 2014, the government-initiated pilot sterilization projects were implemented by 67 out of 345 municipalities. Beginning in 2018, the government released a public bid for proposals for the sterilization of cats and dogs throughout the country, and non-governmental organizations (NGOs) were eligible to apply. From 2018 to 2020, approximately USD 1.5 million had been awarded to NGOs for sterilization. To cover all the costs of sterilization, including staff, CLP 25,000 (approximately USD 35) per animal was awarded to successful applicants. These campaigns were done in communities, usually in a community center rather than in a controlled setting such as a clinic and were offered free of charge to any owner regardless of their economic status. The PTRAC application process states that they place a higher priority for the approval of sterilization programs on those areas with the highest population of pets, with a special emphasis on areas with little to no presence of veterinary services, very remote areas, areas with high numbers of abandoned or stray animals, the lowest income or the highest vulnerability communities, a close proximity to protected wild areas with threatened wildlife species, and regions with a high prevalence of canine zoonoses. It is unknown how some of these parameters, such as numbers of abandoned or stray dogs and the zoonotic disease prevalence was reported, as these data are not available for many communities.

Additionally, the municipalities continued to offer free sterilization and microchip campaigns to the communities through PTRAC, and from 2014 to 2020, they had offered 1183 sterilization projects across the country, sterilizing almost half a million animals. Another component of the veterinary services strategy was to address a deficiency in municipalities with poor technical veterinary expertise and/or access to primary veterinary care in municipalities. Veterinary medical professionals were hired for six months to develop a community strategy for responsible ownership and to implement actions for the application of Law 21.020. In eight communities determined as priorities by the aforementioned criteria, primary care veterinary centers were built.

### 3.4. PTRAC Strategy 2: Education in Responsible Pet Ownership

The education program is designed to promote behaviors that favor the RO of companion animals in citizens ranging from preschool ages to secondary education, as well as the general public, through training, courses, and talks. National funding is available to municipalities and NGOs. The approved projects range from online videos about RO, to virtual programs, to presentations for the public, to in-school talks and activities. Municipalities are also required to hold workshops to disseminate information about RO and Law 21.020, as well as hosting several larger public outreach events throughout the country, such as Pet Pelusa and Protected Pet in your Plaza, which are large, fair-type family events with information stands and activities related to adoption and RO.

**Figure 3:**
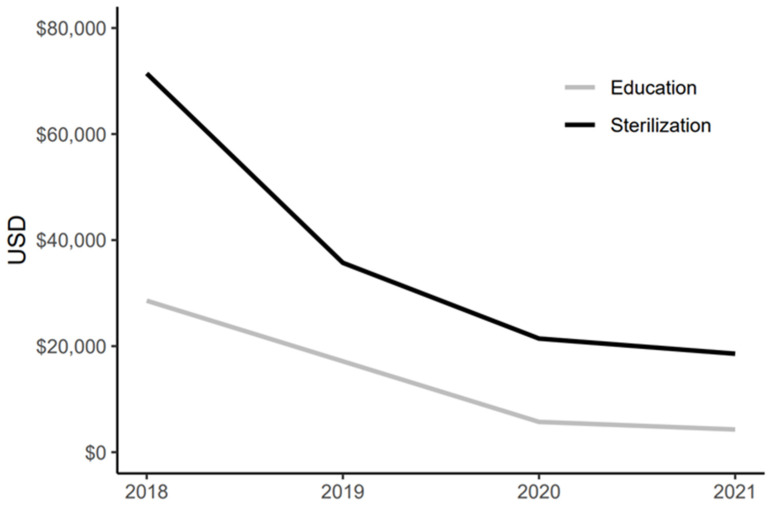
Maximum allowable amounts (CLP) that an organization could be awarded for sterilization and education projects from 2018 to 2021.

From 2016 to 2020, a total of 132 municipal education projects were approved and implemented. Beginning in 2018, NGOs were eligible to apply for funding, and between 2018 and 2020, 45 NGO-managed projects were financed. In 2018, successful education projects were awarded up to a maximum of CLP 20 million (approximately USD 25,000); however, this was reduced in subsequent years (likely attributable to the COVID-19 pandemic) and by 2021, the amount had dropped to a maximum of CLP 3 million (approximately USD 3800) per project, a drop of 15% compared to the original amount. By comparison, the sterilization program also dropped from year to year and is now 26% of the original amount in 2021 (Figure 3) [45].

Between 2017 and 2020, a total of 177 education projects were financed. The government also offered an online course about RO to successful applicants and government employees involved in the program, and although participation was encouraged, it was not required. While each NGO was vetted for prior experience in education delivery and the validity of the proposed project during the proposal stage, there were no requirements to measure and report on the impacts of any of the programs on the target audience. The accountability for awarded funds was based on how the funds were spent and the numbers of children or schools participating, for example, but not on knowledge gained, retention, or changes in attitudes.

Although the PTRAC states that RO education should be delivered under a strategic framework with methodologies that allow for the continuity of the program that achieves significant and extensive learning for the whole community, the entire education budget allocated to NGOs went from approximately USD 240,000 in 2018, to USD 303,000 in 2019, down to only USD 65,000 in 2020 for the whole country, and the total allowable time for which NGOs were allocated to execute their projects went from eight, down to a maximum of four, months.

### 3.5. PTRAC Strategy 3: National Registry

As part of the PTRAC, the plan was to develop five national registries for the following: (1) all cats and dogs in the country, (2) dangerous breeds, (3) NGOs that work in the field of small animal health or welfare, (4) breeders and sellers, and (5) shelters. As of the end of 2019, only the national registries for all cats and dogs and the NGOs were operational [29].

**Figure 4:**
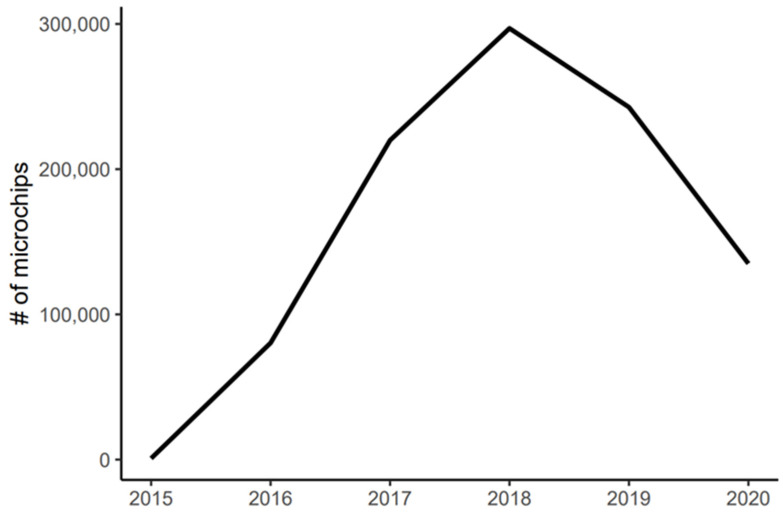
The number of microchipped cats and dogs between 2015 and 2020 shows a steady rise toward 2018 and then a decline into 2020.

The deadline for compliance with Law 21.020, stating that all dogs and cats required identification through internal or external means, was 12 February 2019. The accepted means of identification include a microchip for internal identification, and a tattoo or a collar with identification tags containing a unique code designated by the government is accepted for external identification. From 2015 through to 2018, there was a steady increase in microchipping through the PTRAC program, possibly as a result of the national public outreach campaign or, alternatively, because the implantation with a microchip was obligatory for any animals sterilized at any of the PTRAC campaigns (Figure 4). One year after registration became obligatory, there were 969,579 dogs in the database [46] and on 13 February 2021, there were 1,218,182 dogs registered [47].

## 4. Discussion

Although the draft law for responsible pet ownership was frozen in the Chilean parliament for many years, it was thanks to public pressure that the Law of Responsible Pet Ownership (Law 21.020) in 2017 was finally approved. Before this date, pets were considered “property” and now they are considered sentient beings requiring certain standards of care, health, and management. The government then moved to the next step to develop a companion animal program (PTRAC) and allocated significant public funds toward its implementation. They have dedicated time and money to seek public and municipal buy-in and have had great success with the numbers of municipalities that are participating in the program, stretching from the north to the south of the country. Despite the country experiencing significant social unrest in 2019 and then the pandemic in 2020 and 2021, resulting in the available funds being greatly reduced, the government has continued to make DPM a priority. As with any new program, there are a number of pitfalls that could be addressed to greatly improve the efficiency and impact of the PTRAC. Below, we discuss some of these and suggest a few alternative possibilities for consideration.

### 4.1. PTRAC Design

In published recommendations for the design of a DPM program, the most critical step is to do an assessment of the human perception of the problem, pet demographics, and a local needs and resources assessment within a geographic area [8,9]. This information provides the basis from which to form appropriate objectives, justify the chosen strategy, and to evaluate progress. National objectives can then be tailored to represent the local reality, indicators can be chosen that correctly align with the objectives, and funds can then be allocated appropriately. Since the overall objective of the PTRAC was to improve the quality of life of the people, the interventions should have been linked to a related human parameter. A key measurement of success could then have been of a more sociological or anthropological nature (such as qualitative and quantitative surveys and focus-group interviews) to evaluate whether members of the public experienced the relevant attitudes, perceptions, or behavior changes towards dogs in their communities.

For example, in some communities the primary concerns may be the fecal or garbage contamination of public spaces. The public perception of change, following targeted interventions such as the availability of dog-specific parks with fecal collection bags, the presence of enforcement officers, and/or dog-proof garbage containers, for example, would then be important and appropriate indicators.

In a study in remote Australia, the community members’ perceptions about dog conflicts were documented, and the researchers found that they were not as interested in how many dogs there were. Rather, community members sought a balance between the services that the FRDs provided to their community (i.e., protection) and the negative public health effects, including bites and attacks on humans, zoonoses, and poor community hygiene due to dog feces and garbage [48]. This highlights the importance of preliminary consultation with the public to elucidate the problems and then select appropriate solutions based on the local need.

In terms of the Chilean PTRAC, baseline information would have been valuable to assess what strategies were actually needed. For example, in one city of approximately 145,000 inhabitants in south-central Chile, a subsidized municipal veterinary clinic was erected to provide free services. According to the College of Veterinarians for this region, the city held 30–40 small animal veterinarians registered in the College of Professional Veterinarians (Pers. Comm. César Bauza, President, 25 August 2021), an unknown number of unregistered veterinarians (registration with the College is not obligatory in Chile), and a veterinary college (Universidad Austral de Chile, Valdivia, Chile) with specialty clinic services. According to an article on veterinary economics [49], a city in the United States of 40,000 people could support between 3.1 and 4.9 full-time veterinarians. Although there are certainly economic and cultural differences across the two countries, this gives us some indication that the local market could very likely have been over-saturated with veterinarians even before the new municipal clinic was built.

Indicators were developed to evaluate the PTRAC; however, the lack of a clear and direct linkage between them, the objectives, and the goals, severely hindered the ability to measure successes in each of the PTRAC strategies [29]. SMART goals (Specific, Measurable, Attainable, Relevant and Time-bound) are a useful way to clearly determine if the project is on track for success and to select indicators for accomplishing each goal [50]. The presentation of the numbers of animals treated or sterilized, the amount of money spent, or the number of municipalities participating, while it does show a point-in-time intervention, does not necessarily constitute success [31]. Arguably, the cost of collecting baseline data in every community prior to initiating interventions is high; yet, when we look at the amount of money spent on average per year over the last eight years (approximately USD 5,125,000), perhaps one year’s worth of funding could have been disbursed among the 345 municipalities for an average amount of almost USD 15,000 per municipality to do an initial assessment prior to the intervention. This would have greatly facilitated the ability to determine the needs at the community scale and would have empowered local municipalities to develop a more relevant program that can be monitored over time.

### 4.2. Public Health Impacts

Although the Chilean public was listed as the main beneficiary of the PTRAC, and free-roaming dogs have been reported as posing a significant risk to the public affecting the quality of life for the citizens of Chile [29], it is surprising that the public health effects of FRDs do not play a central role in the program. Often, DPM managers jump immediately to sterilization as a silver bullet approach for resolving human–dog conflict; however, the sterilization of pets does not necessarily equate to a reduction in human-directed dog aggression [51]. Dog attacks and bites pose a significant risk to the public in Chile and in many other countries [52,53,54] and is relatively easy to monitor over time; a positive outcome for the public would be to see a reduction in the reported bites per year. In Chile, cases reported to emergency rooms have actually risen from 33,579 in 2018 to 47,290 to 2019, and then to 45,045 bites in 2020 (source: https://reportesrem.minsal.cl/ (accessed on 2 September 2021)). Between 2003 and 2012, it was estimated that bites caused by dogs may have cost the national healthcare system as much as USD 177.9 million, and this estimate does not factor in losses due to the burden of disease borne by individuals in the community, such as a loss of income, and disability [55]. Unfortunately, the public database does not specify any information about the offending dogs, for example, whether it was owned by the victim or whether was an unknown street dog. To permit a more accurate evaluation of the program effects on public health, this would be essential information for determining whether the bite occurred from the victim’s own dog or from an FRD.

### 4.3. Dog Population Estimates

The human-to-dog ratio of 5.1:1 was used across the entire country to estimate the owned dog population. Two immediate concerns arise in regard to the universal use of this ratio. Firstly, estimates were taken exclusively from urban municipalities only in central Chile in 2002, failing to consider the differences in rural areas and in the rest of the country. Secondly, they were related to human population estimates from 2017. This method fails to account for the known differences that can be found in human-to-dog ratios between rural and urban areas across the country, as well as the temporal changes in the human population between the time the human-to-dog estimates were measured and the human population values they relate to.

In Latin America, we found reported ratios of humans to dogs ranging from 3.4:1 and 4.3:1 in Mexico [56,57], 4.0:1 in Brazil [58], 4.6:1 in Bolivia [59], and 7.6:1 in Ecuador [60]. In other parts of the developing world, detailed studies reported differences between rural and urban areas; for example, in Africa they reported an average of 2.1:1 in urban areas and 7.4:1 in rural areas [61], while Asia reported averages of 7.5:1 in urban areas and 14.3:1 in rural areas [61].

Chile has published ratios of humans to dogs that are similar to these other countries, such as 6.4:1 in Santiago [36], 5.2–6.2:1 in the city of Coquimbo [5], and as low as 10.2:1 and 10.7:1 in El Carmen and Los Angeles, Chile, respectively [35]. However, other published articles found that in some regions of the country, a very high human-to-dog ratio was revealed, with some studies reporting ratios of 2.3–5.3:1 in towns near Coquimbo [5], as well as ratios as high as 1.1:1 to 2.1:1 in rural areas near Coquimbo [5] and 1.3:1 in rural areas near Valdivia [38]. To our knowledge, these would be some of the highest reported human-to-dog ratios in the world.

Interestingly, the dog population estimate did not appear to have been used to determine community-specific interventions and goals; rather, it seems that the same strategies were applied across all sites, independent of the sociocultural uniqueness of communities, whether the site was rural or urban, and what the dog population estimate was. A local needs assessment would have greatly aided in determining the requirements for technical capacities, the accessibility of veterinary care, sterilization or microchipping, and priority areas for disease control and the control of dog abandonment. With a clear picture of the differing community-specific needs, public funds could have been allocated toward appropriate strategies and priority areas.

Here we presented a statistical tool (a model) that allows for more reliable estimates of these ratios to be derived for municipalities where human density is known, but the total number of dogs in unknown. Using this model, we can predict the ratio of humans to dogs for a given human density, resulting in an estimate that is more accurate than the default 5.1 ratio commonly used across the whole range of human densities in the country.

### 4.4. Model Caveats

It is important to note that, given the relatively large variability of the ratio in the dataset at all levels of human density, the model achieves a moderate level of precision in its predictions. However, considering that the current default value (5.1), which essentially assumes a constant ratio for all municipalities, the outputs of this model constitute an improvement in the estimation of more accurate values of the ratio of humans to dogs. The model is a particularly useful tool in municipalities with lower human densities (below approximately 100 hab/km^2^), where the confidence interval does not overlap with the default 5.1 (see Figure 2).

It is also worth noting that this model describes a pattern between human density and the human-to-dog ratio at a broad municipality scale, which is not able to account for the possible differences in this relationship between urban and rural areas within a municipality. This limits its applicability for more fine scale estimates of the ratio, such as in small communities that wish to estimate the ratio as a baseline using human density measured in their locality. Unfortunately, there currently are not enough data available at the small locality scale with corresponding human densities. Thus, we recommend that an actual census be done, or that this tool be applied only at the municipality level, until more fine scale data becomes available, allowing for a reformulation of the model.

### 4.5. Sterilization of Dogs

A reduction or stabilization of the dog population is a goal that planners might quickly jump to, but its feasibility and necessity come into question. Although surgical sterilization is a permanent solution to the reproduction of the individual dog, it is important to carefully consider what the objectives of a reproductive control strategy are. For example, it is suggested that at least 70% of the female population, both owned and unowned, must be sterilized [2] in order to attain a reduction in the population. However, this number varies according to demographics, such as the turnover rates of the target population [1]. For example, in the Bahamas, it was found that 83% of the females were required to be sterilized in order to reach a population equilibrium [62]. This kind of a goal is difficult to reach and maintain, and, in fact, it is a goal that few countries have been able to achieve [1]. For example, in Italy, FRDs were trapped in the streets and sterilized over a period of 14 years. It was assumed that over this period of time, all the dogs would be sterilized and the FRD population would experience a progressive aging and disappearance. However, this is not what happened. It was determined that the rate of immigration was higher than the rate of sterilization, so unfortunately, the goal of controlling the FRD population size through sterilization was never achieved [6]. Additionally, studies of sterilization programs suggest that the goals of reducing a population through sterilization will be met anywhere from 10 to 30 years, depending on the local conditions and the sterilization effort [62,63]. These examples of challenges and failures in dog population control highlight the importance of understanding the local population and the need for site-specific management to reduce the ineffective spending of public funds and to increase the probability of success.

In Chile, the PTRAC does not specify what the goal of sterilization is in any of the 16 regions of the country. Using the PTRAC human-to-dog ratio of 5.1:1, Table 1 shows the sterilization totals by region and an approximate sterilization rate of 6.32% was reached across the country without taking into account dogs sterilized during the same time period in private clinics. Without having a more precise number of dogs in the country, the impact of the sterilization campaigns, therefore, is difficult to interpret. Iit is likely too low to have a population effect. Perhaps a more specific and meaningful goal, and one that aligns better with the concept of responsible ownership, is to strive for a higher level of the humane containment of owned animals. Focusing on education about RO, humane dog containment, and the sterilization of owned pets may bring about a cultural change that is reflected by fewer unsupervised dogs roaming the streets.

### 4.6. Free Veterinary Services

Responsible dog ownership is a critically important part of any DPM program, as it has the potential to reduce community and environmental conflicts, canine diseases, zoonotic diseases, and to improve welfare in the long term. Pet owners must understand that their animal’s actions, health, and welfare are their responsibility and not that of the government or local NGOs, otherwise there is a very high potential for the program to fail. The PTRAC strategy of offering free services to community members, such as veterinary services and sterilization campaigns, runs the risk of creating a dependency by teaching owners that the responsibility for the veterinary care of their pets lies in the hands of the government. The goal of offering free veterinary services at municipal campaigns is contradictory, because the PTRAC states that taking full responsibility for pets is an obligation of all pet owners.

Free municipal clinics also have the potential for creating unnecessary competition with privately owned veterinary clinics in the same community. Many clinics use their spay/neuter income as their most important service item for paying overheads [64]. In the United States, low-cost spay/neuter clinics are common DPM strategies, and while some clinics initially perceived them to be a threat to their business, a survey in 2012 [49] demonstrated that there was actually no decrease in clinic income. In fact, there was a positive relationship between the presence of these campaigns and the number of sterilizations done in private clinics [65]. The authors attributed this to the fact that the benefits and importance of the sterilization of pets were broadly advertised as part of the campaign marketing, along with exerting public pressure to be responsible owners. At the same time, the discount is only offered to a segmented market by setting stringent income restrictions for the receipt of services [65], so those belonging to a higher economic class would be required to pay the full cost in a private clinic.

It is important to note that the above discussion refers to low-cost campaigns, not free campaigns, as is currently being offered in Chile. Underserved communities and low-income populations are a neglected demographic both in terms of human and animal health [66] and it is of utmost importance that these communities are provided with the means to access veterinary care. However, free services may not be the solution. In one source, the provisioning of free services has been termed toxic charity [67]. This term describes the unintentional disempowerment of communities through the charitable giving of things that they are perfectly capable of paying for. There are other models, such as sliding scale payments or low-cost campaigns, that are more likely to improve outcomes, such as emphasizing important concepts about who is responsible for pet care (the government versus the owner), as well as enhancing the dignity of community members receiving charity [67]. Dog owners and caregivers need to understand and appreciate the importance and true costs of veterinary care to ensure that they engage with this service in the long term. Free veterinary care should only be considered with great care and under exceptional services, such as emergency outbreaks [9].

We suggest that in Chile, a government household income-based classification system be used to determine the level of household vulnerability (e.g., Social Registry for Households/Registro Social de Hogares) to determine whether a pet owner is eligible to receive low-cost services. In communities that are determined as having a sufficient number of private clinics for the pet-owning population, the low-cost services could even be offered through these existing private clinics as an additional service. In this scenario, owners eligible for a subsidy would be required to pay a percentage of the cost, and the government could contribute the remainder. This would serve multiple purposes; for example, it would reduce the need for additional and costly brick and mortar municipal clinics in places where there are already sufficient services, as well as reducing the need for additional veterinary surgeons to be employed in every municipality. Spay/neuter campaigns could be offered in existing clinics, rather than community centers, and this would allow for surgeries to be performed under aseptic conditions with the correct equipment, supplies, drugs, and personnel nearby should there be an emergency. Lastly, it would engage local veterinarians and involve them in the PTRAC program, allowing them to have enhanced opportunities to interact with, and educate, all sectors of the community about responsible ownership. This opportunity could lead to longer-term relationships between the government and private sector veterinarians, and between pet owners and their veterinarians. It would also release an enormous amount of public funds to support other areas of the program, for example, pre-program assessments, baseline date collection, education, and enforcement.

### 4.7. Identification and Registration

The identification and registration of dogs is the foundation of any DPM program to create compliance and sustainability. However, if the data are stored in a registry and there is no active enforcement program in place, the full effect of the activity is nullified. An important requirement in the process of developing a new public policy is to evaluate the willingness of the public to comply with the law and the ability of local municipalities to enforce the new regulations [68]. It is not sufficient to simply publish the new law and expect the public to know about it and voluntarily comply with it [69]. The purpose of enforcement is to ensure the compliance of the law and to exact an eventual modification of individual and cultural behaviors. Governments generally rely on the “deterrence paradigm” in which people fear sanctions and penalties and will eventually change to avoid them [70].

If a government wants to improve regulatory compliance, it is critical that it first understands the obstacles to compliance, what motivates the target individuals to comply with the regulation, and what the target group is doing in real life [69]. This should be part of the initial citizen participation period and should include a practical assessment of the ability of the municipalities to enforce the law, and if not, determine ways in which this kind of support can be delivered to communities.

Once the public is aware of the new law, voluntary compliance is seldom sufficient to incite permanent change and a key determinant of government effectiveness is how well they achieve their policy objectives [69]. To put the responsibility on neighbors and community members to report infractions is relying purely on the trust and honor system, which is not an efficient manner of enforcing new regulations and moving toward a permanent cultural and behavioral shift [69].

In fact, in a study in remote Australia, it was found that survey respondents were loath to confront their neighbors or call the enforcement department regarding dog-related complaints, and instead chose a “weary acceptance and passive resentment” that tended to create underlying social tensions in the neighborhood [48]. In the case of the Chilean government, they seem to rely on this passive type of enforcement, or in other words, relying on community members to report observed infractions to the authorities, rather than having enforcement officers actively patrolling neighborhoods and delivering fines to offenders. In the city of Valdivia, in south-central Chile, for example, from the beginning of 2017 to 13 July 2021, only 77 fines had been issued over 4.5 years to owners of dogs for violating the law (source: Municipality of Valdivia, accessed on 7 June 2021) and 292 over a two-year period (2017 and 2018) in the whole country [29].

As part of Law 21.020, there is a fine for owners whose pets are not identified and registered. Initially, only permanent identification (i.e., a microchip) was deemed acceptable, but this was later changed to include collars with identification tags as acceptable due to public pressure. Collars and tags used alone, however, are not reliable, as they are easily removed, applied to other animals, and pose a potential danger for FRDs that can become caught or hung up on fences, for example [71]. While an increase in microchipping was observed from 2015 to 2018, the numbers have declined steadily since then (Figure 4). Based on the estimated human population in 2021 and the estimated owned dog population, using the 5.1:1 ratio (see Table 1), approximately 25% of owned dogs are microchipped and only 31% are registered in the national database. While the numbers of registered dogs continued to climb slowly from 2018 to 2021, the microchipping rate dropped drastically after 2018 (Figure 4). Since many municipal campaigns were cancelled during the COVID-19 pandemic, fewer microchips have been placed by the government, and while the government encourages microchipping, owners appear to be choosing external identification over a permanent microchip. This is concerning, as a collar and tags are not permanent means of identification; thus, they negate the utility of an identification program. Furthermore, the rate of registration, whether by permanent or temporary identification, is still low. Perhaps this is due to a lack of enforcement of Law 21.020 in communities. This may be interpreted by the public as being of low priority by the government, resulting in people concluding that the risk of penalty is negligible and, therefore, placing a low importance on owners to identify and register their pets.

Perhaps, in those communities where there was already sufficient veterinary infrastructure, a better strategy other than investing in sterilization campaigns or building municipal clinics could have been to build enforcement teams of animal control officers, for example. Having enforcement personnel on the ground in these communities, although it will always be opposed by some, can also be very validating for community members that are asking for visible action. Their perception of the problems and the associated actions taken by the municipality as the program is unrolled can be an important indicator to evaluate whether the program has the desired effect of people and pets living together in a more harmonious manner.

The enforcement of legislation can strongly support community efforts to manage dog-associated problems and it is a critical element for the sustainability of the program as a whole. Fines and registration fees can also contribute to the long-term sustainability of the program [1]. Sadly, all community and government efforts can fail if enforcement is not seen as a priority [1]. Full time animal control officers can greatly assist in the delivery of the program, not only through the administration of fines, but also through the registering of pets and performing annual street dog surveys and/or household surveys, as well as raising public awareness about the law and PTRAC. Through these ongoing activities, governments can set reasonable and achievable indicators for their end goals, such as annual trends of numbers of registered animals, the number of fines given out, the numbers of sterilized versus intact animals being registered, and the numbers of FRDs in the streets.

### 4.8. Responsible Pet Ownership Education

Enforcement alone is not considered an effective enough tool to change human behavior, and other strategies, such as education, are used in combination to attain the desired change [72]. The PTRAC education program’s long-term objective is to provoke a change in current and future generations. Therefore, it is critical that they are monitored, evaluated, and then adapted based on feedback so that the interventions have an impact in increasing knowledge and awareness, or an actual change in human behavior, for example. Yet, the education recipients were not evaluated for any of the above parameters. A more effective method of delivering a national education program might be to have a professional education curriculum and outreach package developed by a team of educators and animal healthcare providers where grants can be awarded to NGOs to deliver the standardized program and evaluation packages to schools or the public. This approach would ensure the maintenance of the quality of the content and the alignment of the message to children of all ages, across the entire country, similar to a school curriculum. Simultaneously, obtaining government cooperation to include a certain number of hours of RO teaching, that is required in the national school curriculum per year, would aid in the delivery to children and provide a more supportive approach to obtaining compliance at the school level, rather than relying solely on the individual teachers’ interest in the topic.

In the case of Chile, there was no budget allocated from the program for the enforcement of the Cholito Law, and the entire education budget allocated to NGOs went from approximately USD 240,000 in 2018, to USD 303,000 in 2019, down to only USD 65,000 in 2020 for the whole country. One of the main goals of the program is to improve the human relationship with pets. This has been successfully demonstrated through RO education programs [73,74,75], but if there is no budget allocated toward enforcement and very little toward education, it is unlikely to be successful in provoking a sustained behavioral or cultural shift in society.

## 5. Conclusions

Although the Chilean government has made great strides in bringing forward a law to protect companion animals and a program to reduce conflict between pets and the public, the strategies chosen to achieve this goal have some fundamental flaws. Specifically, we recommend reassessing the PTRAC program to include the collection of baseline data at the community level. An analysis to assess the existing resources and needs, and a dog census or population estimate, should be the minimum baseline data required prior to any intervention. In communities where a dog population estimate is logistically or economically impossible, a more specific statistical tool, such as that included in this article to estimate the dog population based on human density, should be used. In each community where a DPM program is designed, we recommend the development of appropriate intervention strategies and SMART goals, as well as the use of some simple indicators that are all based on the initial baseline data results for each community. This would greatly assist managers in determining whether goals are being met and could lend more accountability for the use of public funds. Fund allocation should be adjusted according to the prioritization of problem areas based on the initial assessments results and demonstrated need so that funds are used prudently. The delivery of free veterinary services should be eliminated and, instead, the government could consider low-cost alternatives. Funds that would otherwise have been spent on free services could then be invested in long-term strategies aimed at human behavior change such as education and enforcement.

## Figures and Tables

**Table 1:** Human populations by region of Chile in 2021 and estimated dog population using the PTRAC default of 5.1 humans per dog. Note that the total number of dogs sterilized between July 2017 and the end of August 2020 was 243,915 (https://www.biobiochile.cl/noticias/nacional/chile/2020/08/08/1-3-millones-mascotas-registradas-449-mil-esterilizaciones-3-anos-aprobada-la-ley-cholito.shtml (accessed on 3 January 2022)) resulting in a presumed nation-wide sterilization rate of 6.32%.

Region	Estimated Human Population 2021	Estimated Owned Dog Population Based on PTRAC 5.1:1
Metropolitana de Santiago	8,242,459	1,616,168
Valparaíso	1,979,373	388,112
Biobío	1,670,590	327,567
Maule	1,143,012	224,120
La Araucanía	1,019,548	199,911
O’Higgins	1,000,959	196,266
Los Lagos	897,708	176,021
Coquimbo	848,079	166,290
Antofagasta	703,534	137,948
Ñuble	514,609	100,904
Los Ríos	407,837	79,968
Tarapacá	391,558	76,776
Atacama	316,168	61,994
Arica y Parinacota	255,068	50,013
Magallanes y Antártica Chilena	179,533	35,203
Aysén	107,158	21,011
Total	19,677,193	3,858,273
Total dogs sterilized between July 2017 and August 2020		243,915
Percentage of dogs sterilized at country level since the approval of Law 21.020		6.32

## Data Availability

The data presented in this study are openly available in FigShare at https://figshare.com/s/a35908be20e8d0d2018a (accessed on 8 December 2021).
